# Measurement of maternal morbidity during postpartum with the WHO-WOICE tools in Morocco

**DOI:** 10.1186/s12884-023-05615-4

**Published:** 2023-05-02

**Authors:** Hanane Hababa, Bouchra Assarag

**Affiliations:** National School of Public Health, Rabat, Morocco

**Keywords:** Maternal morbidity, Measurement tools, Postpartum care, Mental health, Sexual health

## Abstract

**Background:**

Maternal morbidity refers to any health problems or complications experienced by a woman during pregnancy, childbirth, or the postpartum period. Many studies have documented the, mostly negative, effects of maternal ill-health on functioning. Although, measurement of maternel morbidity remains underdeveloped. We aimed to evaluate the prevalence of non-severe maternal morbidities (including overall health, domestic and sexual violence, functionality, and mental health) in women during postpartum care and further analyze factors associated with compromised mental functioning and clinical health by administration of the WHO’s WOICE 2.0 instrument.

**Methods:**

A cross-sectional study was conducted at 10 Health centers in Marrakech, Morocco with WOICE questionnaire included three sections: the first with maternal and obstetric history, sociodemographic data, risk and environment factors, violence and sexual health; the second considers functionality and disability, general symptoms and mental health; and the third includes data on physical and laboratory tests. This paper presents descriptive data on the distribution of functioning status among postpartum women.

**Results:**

A total of 253 women averaging 30 years of age participated. For self-reported health status of women, more than 40% reported good health, and just 9.09% of women had a health condition reported by the attending physician. Among postpartum women with clinical diagnoses, 16.34% had direct (obstetric) conditions and 15.56% indirect (medical) problems. When screening for factors in the expanded morbidity definition, about 20.95% reported exposure to violence. Anxiety was identified in 29.24% of cases, and depression in 17.78%. Looking into gestational results, just 14.6% delivered by cesarean section and 15.02% had preterm birth. We found also that 97% reported “good baby health” in the postpartum evaluation, with 92% of exclusive breastfeeding.

**Conclusion:**

Considering these results, improving the quality of care for women requires a multi-faceted approach, including increased research, better access to care, and improved education and resources for women and healthcare providers.

## Introduction

Maternal health refers to the physical and emotional well-being of women during pregnancy, childbirth, and the postpartum period. It involves access to quality health care services, education on pregnancy and childbirth, proper nutrition, and support for the mother and her baby. Improving maternal health is important for reducing maternal morbidity and mortality, and for promoting the health and development of the next generation [1, 2].

According to the World Health Organization (WHO), approximately 830 women die every day from preventable causes related to pregnancy and childbirth. The majority of these deaths occur in low- and middle-income countries, where access to quality maternal health care is often limited [3]. Additionally, many women experience pregnancy and childbirth-related complications that can have long-term effects on their health and well-being. Improving maternal health and reducing maternal mortality and morbidity is a global health priority, and requires a multi-faceted approach that includes strengthening health systems, increasing access to family planning and reproductive health services, and promoting gender equality and women’s empowerment [4].

According to Lale Say et al [2], maternal mortality only captures a portion of the overall burden of maternal ill health, as it excludes maternal morbidity. The burden of maternal morbidity is not yet known [4]. WHO estimates that for every recorded maternal death, 20 to 30 women suffer morbidity. Of these cases, one quarter may suffer severe and permanent sequelae. These sequelae can affect women physically, mentally, sexually, in their ability to function (cognition, mobility, participation in society), body image and socioeconomic status.

The standard criteria for the identification of potentially lifethreatening conditions (PLTC) and Maternal Near Miss (MNM) have helped in improving the understanding of severe maternal morbidity (SMM), but it is now recognized that a broader approach is needed to fully understand maternal morbidity, including non-severe forms of morbidity [2]. This requires a more comprehensive understanding of the health and well-being of women during pregnancy and childbirth, as well as an appreciation of the social, economic, and cultural factors that contribute to maternal morbidity [5]. By taking this broader approach, it may be possible to identify new strategies for improving maternal health and reducing maternal morbidity, which will be important for achieving the Sustainable Development Goals for 2030.

The definition developed by the World Health Organization’s Maternal Morbidity Working Group in 2012 is a significant advancement in our understanding of maternal morbidity. By defining maternal morbidity as any health condition that is attributed to pregnancy or childbirth and has a negative impact on a woman’s well-being or functionality, it recognizes the broad spectrum of morbidity that women can experience, including both severe and non-severe forms of morbidity. In 2012, the World Health Organization (WHO) recognized the lack of standardized instruments for accurate assessment of maternal morbidity and created the Maternal Morbidity Working Group (MMWG) to address this issue [6]. The MMWG created the WOICE (Women’s Outcome Instrument for maternal health Care) instrument for measuring maternal morbidity, which focuses on a woman’s health and self-perception of well-being. The WOICE instrument is designed to address this issue by providing a comprehensive assessment of the health and well-being of women during the postpartum period. Lack of knowledge about such conditions lead to inadequate care of these women and contributes to possible short and long- term consequences [7, 8].

The aim of this study is to gain a better understanding of the health issues that women face during the postpartum period in a middle-income setting. By using the WOICE instrument, the objective of the present study is to assess the prevalence of non-severe maternal morbidity among puerperal women. This will help to identify the common health problems that these women face and to determine the factors associated with impaired clinical, social, and mental health conditions.

## Method

This cross-sectional study used a questionnaire developed by the WHO to assess maternal morbidity, which includes various instruments that measure different aspects of maternal health. The maternal morbidity measurement questionnaire called WOICE was originally developed in English and further translated into the French. The review was conducted by experienced obstetric investigators and the version was tested (pilot interviews) to measure the time of application and then adapt and modify some words to ascertain accurate understanding. This several tools have already been adapted. Adapting these tools to the Moroccan context helps to ensure that the results are relevant and meaningful to the local population. WOICE includes the 12-item version of the World Health Organization’s Disability Assessment Schedule (WHODAS 2.0). This tool evaluates the functionality and ability to perform daily tasks also includes a tool that evaluates mental health, the General Anxiety Disorder 7-item test (GAD-7), and the 9-item Patient Health Questionnaire (PHQ-9), to assess depression, both already adapted [9, 10]. To measure sexual satisfaction and sexual and domestic violence, parts of some scores already validated are within the WOICE, such as the Brief Sexual Symptom Checklist for Women (BSSC-W) and some questions from a questionnaire used in the Multicountry Study on Women’s Health and Domestic Violence against Women of the WHO [11]. For substance use and abuse, WOICE includes Alcohol, Smoking and Substance Involvement Screening Test (ASSIST) [12, 13]. We confirm that all methods were performed in accordance with the relevant guidelines and regulations.

The final pilot PPC questionnaire includes three sections: (1) woman’s history, (2) current symptoms, and (3) a physical examination, including a brief review of her medical records, where available [2]. To describe the different types of morbidity, and stratification by setting, sample size was estimated in 253 participants for convenience sample (156 in Urban and 97 in Rural). Women were recruited sequentially according to their scheduled postpartum visit, during the data collection period. Inclusion criteria were that women were 6–12 weeks after delivery. This study was conducted in 10 health center in Marrakech Morocco, Efforts were made to include 05 centers in rural and 05 in urban. The Ethics Committee of the Faculty of Medicine and Pharmacy of Rabat approved this study. For the administration of the questionnaire, each interview lasted between 15 and 30 min in total, and the physical examination took between 10 and 15 min [2]. All women with age higher than 18 years that agreed to participate signed an Informed Consent form before interview. The questionnaire was always performed after the scheduled medical consultation and with no interference in the woman’s medical follow-up.

Data collection was supported by tablets (Samsung Galaxy Tab Tablets S3 – Android), with further transmission, verification and storage of data protected to ensure confidentiality. Using tablets for administering tools can help improve data quality. Additionally, using tablets can also increase the speed and efficiency of data collection, as data can be entered directly into the device without the need for manual transcription to a separate system. The database was exported to a format compatible with the statistical.

package SPSS for analysis. The data collectors are trained to provide accurate information to women they are conducting assessments with, and to refer them to appropriate services if necessary. They are trained on the different types of services that are available, as well as the criteria for determining when a referral is necessary.

## Results

This study was conducted in 10 health centers, where 253 women were invited to participate. Among the characteristics of our population, the mean age was 30 years (29.78 in urban and 29.77 in rural), just two women (2%) without a partner, and more than 80% were multiparous. More than 90% of the women were not employed. The majority of our study population had a Primary or less school level, and just 4.35% had a higher educational level (Table 1). Over one third of the population cannot read (51.55 in rural and 39.53 in urban), and just 8.70% (4.49 in urban and 15.46 in rural) took more than 60 min to arrive from their house to the health service (Table 1).

**Table 1:** Characteristics of postpartum women (PPC)

Postpartum care	Urban (n = 156) (%)	Rural (n = 97) (%)	Total (n = 253) (%)
**Maternal Age in Year**	29.78 ± 6.00	29.77 ± 6.23	29.77 ± 6.08
< 20	3 (2)	2 (2)	5 (2)
20–34	101 (65)	61 (63)	162 (64)
≥ 35	52 (33)	34 (35)	86 (34)
**Marital status**			
Bride	156 (100)	95 (97.94)	251 (99.21)
Without a husband	0 (0.00)	2 (2.06)	2 (2)
**Level of education**			
Primary or less	104 (66.67)	81 (74.25)	185 (73.13)
Secondary	43 (27.56)	14 (14.43)	57 (22.53)
University	9 (5.77)	2 (2.06)	11 (4.35)
**Literacy**			
Cannot read	50 (32.05)	50 (51.55)	100 (39.53)
Can read part of a sentence	72 (46.15)	29 (29.90)	101 (39.92)
Can read the whole sentence	34 (21.79)	18 (18.56)	52 (20.55)
**Employment**			
No	142 (91.03)	97 (100)	239 (94.47)
Yes	14 (8,97)	0 (00)	14 (5.53)
**Time from C/S to home, min**			
< 15	61 (39.10)	30 (30.93)	91 (35.97)
15–30	52 (33.33)	29 (29.90)	81 (32.02)
30–60	36 (23.08)	23 (23.71)	59 (23.32)
> 60	6 (4.49)	15 (15.46)	22 (8.70)
**Parity**	2.76 ± 1.10	2.68 ± 1.36	2.73 ± 1,21
1	22 (14.10)	14 (14.43)	36 (14.23)
2–4	127 (81.41)	73 (75.25)	200 (76)
≥ 5	7 (4.48)	10 (10.30)	17 (6.71)

*For self-reported health status of women*, more than 40% reported good health, and just 9.09% of women had a health condition reported by the attending physician. Based on the information provided, a list of conditions, classified them as direct and indirect, of which 3.11% had chronic hypertension, and operative wound infection (4.66%) (Table 2). Looking into gestational results, just 14.6% delivered by cesarean section and 15.02% had preterm birth. We found also that 97% reported “good baby health” in the postpartum evaluation, with 92% of exclusive breastfeeding (Table 3).

**Table 2:** Health status reported by women during postpartum care

	Urban	Rural	Total
**Postpartum Care**	**(n = 156)**	**(n = 97)**	**(n = 253)**
**General health status**			
Very good	3 (1.92)	2 (2.06)	5 (1.98)
Well	64 (41.03)	52 (53.61)	116 (45.85)
Neither good nor bad	67 (42.95)	29 (29.90)	96 (37.94)
Bad	19 (12.18)	13 (13.40)	32 (12.65)
Very bad	3 (1.92)	1 (1.03)	4 (1.58)
**Have you been told you have a problem/complication?**			
No	141 (90.38)	89 (91.75)	230 (90.91)
Yes	15 (9.62)	8 (8.25)	23 (9.09)
**Any preexisting conditions**			
No	58 (37.17)	36 (37.11)	94 (37%)
Yes	88 (56.41)	71 (73.19)	159 (63)
**Leading direct preexisting conditions**			
Gestational diabetes	30 (19.23)	12 (12.37)	42 (16.6)
Gestational HT	64 (41)	18 (18.55)	82 (32.41)
Pre-eclampsia	16 (10.25)	5 (5.15)	21 (8.3)
**Number of complications diagnosed**			
0	133 (85.25)	55 (56.70)	188 (73.15)
1	18 (11.53)	24 (24.74)	42 (16.34)
2	8 (7,92)	15 (15.46)	23 (8.94)
3–6	1 (0.64)	3 (3.09)	4 (1.55)
**Categories of complications**			
Direct	7 (4.48)	9 (9.27)	42 (16.34)
Indirect	18 (11.53)	22 (22.68)	40 (15.56)

**Table 3:** Perinatal outcomes, clinical conditions and overall conditions considered by the WHO- WOICE among postpartum women

Postnatal care	PPC N = 253	(%)
**Preterm delivery**		
No	215	84.98
Yes	38	15.02
**Healthy baby**		
No	8	3
Yes	245	97
**Breastfeeding**		
No	20	92
Yes	233	8
**C-section**		
No	216	85.4
Yes	37	14.6

About pre-existing condition, a high percentage of women (63%) reported having a condition before pregnancy and childbirth (Table 2). We found that 16.6% had gestational diabetes, 32.41% had gestational hypertension, and just 08.3% reported having preeclampsia. Looking into use of substances, the majority of participants didn’t use any type of substances during pregnancy. Using the WOICE tool to explore sex life, just 15.02% (11.54% in urban and 20.62% in rural) of the women felt they were satisfied with their sex lives. Problems related to interest in sex after childbirth is the primary reason for sexual dissatisfaction (84.98%) (Table 4).

**Table 4:** Social and sexual conditions among postpartum women

	Urban	Rural	Total
**Postpartum care**	**(n = 156)**	**(n = 97)**	**(n = 253)**
**Exposure to violence**			
Yes	24 (15.38)	32 (32.98)	94 (37.15)
No	132 (84.62)	68 (70.10)	29 (29.90)
**Sexual Satisfaction**			
No	138 (88.46)	77 (79.38)	215 (84.98)
Yes	18 (11.54)	20 (20.62)	38 (15.02)
**Reasons for sexual dissatisfaction**			
Problem related to interest in sex	139 (89.10)	76 (78.35)	215 (84.98)
Problem related to the decrees of the genital sensation	17 (10.89)	26 (26.80)	43 (16.73)
Vaginal lubrication problem	0 (-)	2 (2.06)	2 (0,79)
Problem with orgasm	14 (8.97)	18 (18.55)	32 (12.64)
Dyspareunia	43 (27.56)	24 (24.74)	67 (26.48)

In order to investigate exposure to domestic and sexual violence, we asked participants “whether or not they were afraid of the current partner/ most recent spouse or any other person” if the spouse/ or any other person who pushed, hit and kicked”. About 21% of women reported to have suffered violence (Table 4). We identified, through using the validated scales (PHQ-9 and GAD-7), that 29.24% in this group of women had anxiety symptoms, followed by 17.78 with depressive symptoms (Table 5).

**Table 5:** Mental and functional conditions of the study population

Postpartum care	PPC N = 253	(%)
**Anxiety**		
Yes	74	(29.24)
No	179	(70.75)
**Depression**		
Yes	45	(17.78)
No	208	(82.21)

## Discussion

This study provides valuable insights into the health status during postpartum period in a middle-income setting in Morocco. The use of the WHO-WOICE 2.0 instrument during postpartum care allows for the identification of health problems that may not have been detected otherwise. WHO conducted a pilot study for the first time, in three different countries to test the WOICE in pregnant and postpartum women, with a total sample of 1490 women [1, 2, 6]. Another study conducted for 519 postpartum women in Brazil, and represents the implementation the WOICE 2.0 questionnaire to measure non-severe maternal morbidity for the postpartum women considering many conditions that can impact maternal health [14]. In comparison to results for those studies, our sample included less educated women, older and with partners. In our study, 9.09% of the women reported having a health problem informed by the attending physician. This result is the same as for the pilot study, but much higher (over 50%) in Brazil study. Another marked difference in the current study was a decreased rate of substance use and employment. The distribution of scores between the rural and urban sample were similar, with the exception of direct preexisting conditions which may be in part due to a high detection of risky pregnancies in urban areas.

Our results also showed a greater frequency of exposure to violence, where we could identify that in our group of women surveyed, 20.95% were exposed to some type of violence. The present study also showed that the prevalence of violence varies considerably among women. However, the rates were generally higher (12–14%) than those in the Malawi study (8.4%), and lower than the results of the Kenya study (17.4%). In contrast, the rate of violence in Jamaica was similar (12%). Several studies reported violence against women is a widespread issue that affects millions of women globally. For WHO, 1 in 3 women worldwide suffer from physical and / or sexual partner and sexual violence by third parties at some point in their life. Some studies have shown a prevalence of domestic violence greater than 40%[15, 16]. Violence against women, including physical, sexual, and psychological abuse, is a widespread problem in Morocco. Despite advances in laws and policies aimed at protecting women’s rights, many women continue to face violence and discrimination in their homes, communities, and workplaces. In 2013, the World Health Organization (WHO) estimated that 30% of everpartnered women worldwide had experienced physical or sexual violence by a partner; as had 37% of everpartnered women in the Eastern Mediterranean region, which covers part of the Arab region [17, 18]. These numbers are likely an underestimate, as many cases of violence against women go unreported due to stigma, fear, and lack of access to support services. The results of our study also show that there is a difference in the prevalence of domestic violence between urban and rural areas. This result does not corroborate with a study conducted in Egypt in 2017 [19], which showed that women in rural areas are more exposed to domestic violence than those in urban areas.

Another important aspect evaluated in our study is the sexual health of postpartum women, defined by the WHO as a “state of physical, mental, and social well-being in relation to sexuality” [19, 20]. In our study, self-reported depression and anxiety (using GAD-7 and PHQ-9 for screening) showed a high prevalence of mental disorders (17.78%) compared to the WHO pilot study which revealed rates of 2.2% for postpartum women. According to several studies including this one, many social and economic factors have been associated with postpartum depression, including first pregnancy, and the case of domestic violence [20, 21]. According to a study published in 2016 [22], identifying and treating these women is critical not only for their health, but also for the survival and development of their children. The results of our study corroborate those of two other studies [23, 24] on sexuality during pregnancy and the postpartum period. The first one involved 570 pregnant women, interviewed at T1 (Fifth month of pregnancy), T2 (at 1 month postpartum), T3 (at 4 months postpartum) and T4 (at 12 months postpartum), and showed that at T1 and T2, the majority of women showed significantly less sexual activity and less sexual satisfaction. The second longitudinal cohort study just published in 2018 on 832 prenatal and postpartum women confirms this evidence. Indeed, almost half of the women (46.3%) reported a lack of interest in sexual activity, 43% experienced a lack of vaginal lubrication and 37.5% of the women included had dyspareunia 6 months after birth. The authors of both studies, suggest that practitioners provide family-centered maternity care, they should counsel couples on typical patterns of sexuality during pregnancy and postpartum, and on usual patterns during breastfeeding. Accurate information can help couples feel more comfortable during the transition periods before and after delivery. A discussion of expected changes in sexuality should be always introduced during prenatal care.

Another interesting point of our results is that the majority of women reported good health at the time of the interview. The fact that the majority of women reported good health suggests that they are managing these different aspects effectively, which is a positive sign. However, it’s also important to consider any potential biases or limitations in the data collection method that might affect the accuracy of these results. For example, respondents may not have been completely truthful or may have had different interpretations of what it means to have good or very good health. Further research is needed to understand the full picture of women’s health and to identify any areas for improvement.

Our study has some limitations. The WHO-WOICE instrument evaluates a broad number of aspects. Some are not further detailed, such as violence. Also, the questionnaire is very long, the tools used in postpartum care in Morocco would be reviewed to complement their features with the WOICE aspects. Another limitation is the coordination with general practitioners in order to diagnose morbidities and to follow up on women in order to describe the evolution of morbidities.

## Conclusion

The WOICE-WHO instrument is a tool used to assess the health status of women during postpartum care. The high frequency of anxiety, depression, and violence among women is a major concern and highlights the need for more attention to be paid to women’s mental health during this critical period. Additionally, it’s important for healthcare providers to screen for and identify these issues early on, so that appropriate interventions can be put in place as soon as possible. Improving the mental health and wellbeing of women during postpartum can have long-lasting positive effects on their overall health and the health of their families.

## Data Availability

All data generated or analyzed during this study are available from the corresponding author on reasonable request.

## Abbreviations

PPC: Postpartum care
GAD7: General anxiety disorder 7
MNM: Maternal near miss
MMWG: Working group maternal morbidity
PHQ-9: Patient health questionnaire 9
PLTC: Potentially life- threatening conditions
SMM: Severe maternal morbidity
SPSS: Statistical package for the social sciences
WHO: World health Organization
WHODAS: World health Organization disability assessment schedule 2.0
